# Ultrasound Evaluation of the Combined Effects of Thoracolumbar Fascia Injury and Movement Restriction in a Porcine Model

**DOI:** 10.1371/journal.pone.0147393

**Published:** 2016-01-28

**Authors:** James H. Bishop, James R. Fox, Rhonda Maple, Caitlin Loretan, Gary J. Badger, Sharon M. Henry, Margaret A. Vizzard, Helene M. Langevin

**Affiliations:** 1 Department of Neurological Sciences, University of Vermont, Burlington, Vermont, United States of America; 2 Department of Medical Biostatistics, University of Vermont, Burlington, Vermont, United States of America; 3 Department of Rehabilitation and Movement Science, University of Vermont, Burlington, Vermont, United States of America; 4 Division of Preventive Medicine, Brigham and Women’s Hospital, Harvard Medical School, Boston, Massachusetts, United States of America; Nanjing Medical University, CHINA

## Abstract

The persistence of back pain following acute back “sprains” is a serious public health problem with poorly understood pathophysiology. The recent finding that human subjects with chronic low back pain (LBP) have increased thickness and decreased mobility of the thoracolumbar fascia measured with ultrasound suggest that the fasciae of the back may be involved in LBP pathophysiology. This study used a porcine model to test the hypothesis that similar ultrasound findings can be produced experimentally in a porcine model by combining a local injury of fascia with movement restriction using a “hobble” device linking one foot to a chest harness for 8 weeks. Ultrasound measurements of thoracolumbar fascia thickness and shear plane mobility (shear strain) during passive hip flexion were made at the 8 week time point on the non-intervention side (injury and/or hobble). Injury alone caused both an increase in fascia thickness (*p* = .007) and a decrease in fascia shear strain on the non-injured side (*p* = .027). Movement restriction alone did not change fascia thickness but did decrease shear strain on the non-hobble side (*p* = .024). The combination of injury plus movement restriction had additive effects on reducing fascia mobility with a 52% reduction in shear strain compared with controls and a 28% reduction compared to movement restriction alone. These results suggest that a back injury involving fascia, even when healed, can affect the relative mobility of fascia layers away from the injured area, especially when movement is also restricted.

## Introduction

The thoracolumbar fascia is a prominent anatomical structure in the dorsal trunk region whose role in chronic low back pain is increasingly recognized [1–7]. This thick, multilayered structure is composed of dense aponeuroses that can bear significant loads [4], as well as loose connective tissue layers separating the aponeuroses and allowing shear plane mobility that contributes to the range of motion of the trunk [8]. Recent studies also have demonstrated that the thoracolumbar fascia has a substantial sensory innervation including small caliber nociceptors that can be activated by mechanical stimulation [1, 2, 9]

In a previous human cross-sectional study, we found that subjects with chronic “non-specific” LBP for more than 12 months had both an increase in thickness and a decrease in mobility of the thoracolumbar fascia measured with ultrasound, compared with subjects without LBP [6, 7]. We hypothesized that these structural and functional abnormalities of fascia in subjects with LBP could represent a fibrotic process resulting from an initial soft tissue injury involving fascia, followed by movement restriction that could be worsened by pain or fear of pain. Loss of shear plane mobility between adjacent layers within the thoracolumbar fascia could been one of several interrelated factors in the development of low back pain since the lack of mobility may alter the biomechanics trunk as well as the sensory input (nociceptive and/or non-nociceptive) originating from the fascia. Subjects with low back pain have abnormal motor control strategies that may be in part due to altered proprioceptive input [10], and although the role of fascia in motor control feedback loops is poorly understood, there is evidence that pathological processes involving connective tissue can affect the behavior of overlying muscles [11].

The goal of this study was to test whether an animal model of fascia injury combined with experimentally-induced movement restriction for two months could produce thoracolumbar fascia pathology similar to that observed in human subjects with LBP. Fascia thickness and mobility were measured using ultrasound and ultrasound elastography, respectively, as in our previous human studies [6, 7]. We also tested whether injury and/or movement restriction would produce nervous system neuroplasticity consistent with increased pain sensitivity by measuring spinal cord dorsal horn substance P and calcitonin gene-related peptide (CGRP) expression.

We chose the domestic pig for this study because of its comparable size to humans and substantial similarities in physiology, immune function and wound healing [12–17], as well as skin and subcutanous tissue structure [18]. In particular, pigs and humans are among the few mammals that, unlike mice, rats, rabbits, dogs, and cats, do not have a subcutaneous (pannicular) muscle in the dorsal trunk region, and therefore have a similar relationship between the skin, subcutaneous tissue and perimuscular fascia in the back [17, 19–24]. Movement restriction was induced using a “hobble” device which restricts full hip extension and reduces pelvic lateral flexion in both pigs and humans when walking in the quadruped position.

## Methods

### Experimental Design

The experimental protocols in this manuscript were approved by the University of Vermont (UVM) Institutional Animal Care and Use Committee (IACUC). Castrated male domestic swine (n = 20) between 4–6 weeks old, weighing approximately 4–7 kg were acquired from E.M. Parson’s Hadley, MA. Pigs were group-housed in the UVM animal facility on a 12 hour light-dark cycle and food intake was adjusted to gain approximately 1 lb/day. Animals were trained to stand on scales without restraint to monitor weight weekly. All pigs were exercised daily in a 20’x10’ space for the same amount of time. The remainder of the time, the pigs were allowed to move ad lib in 4’X8’ enclosures. A stabilization period of seven days was allowed before subsequent randomization (week 0) into one of four cohorts: Hobble (n = 5), Injury (n = 5), Injury + Hobble (n = 5) or Control (n = 5) for the duration of eight weeks.

### Gait Analysis

Prior to surgery and/or hobble placement, all pigs including control animals were familiarized with the gait measurement set-up in a controlled environment, weekly over a span of 5 weeks. The apparatus consisted of a PVC tube totaling 3.048 meter (10 feet) in length with evenly spaced floor markers demarcated every .3048 meters (1 foot). Gait measurements were acquired in trials; with one trial representing a distance of 1.626 meters (6 feet) out of the entire PVC tube length. Pigs were trained to walk on a rubber mat alongside the PVC tubing with hobbles removed (if present) during training and also during data collection. At week 7, a maximum of 7 trials (1 trial = 1 lap by 6 markers) per pig were videotaped. Videos were downloaded and analyzed using VideoPad Video Editor Version 2.41. Gait speed was calculated by dividing the distance (6 feet) by the time duration between the pig’s first foot entering past the first marker and the last foot passing the final marker. Stride length (feet) was determined by observing the distance from where the pig set down its front foot in relation to the distance on the bar to when it set the same front foot down again. Counting for number of steps (defining “step” as the moment that either right or left front foot touches the ground) began when the first front foot was placed near/on the first marker, and ending when one of the front feet passed the end marker.

### Movement Restriction (Hobble)

**Fig 1:**
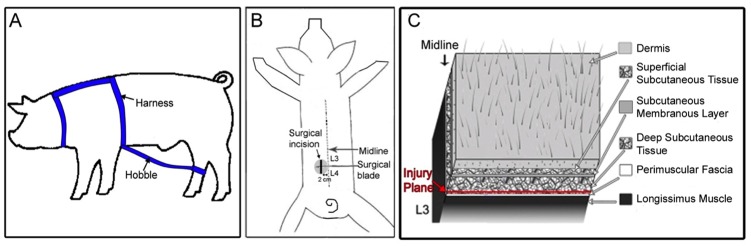
Movement restriction and fascia injury methods. (A) Hobble device used to induce movement restriction. (B) Location of fascia injury. (C) Location of fascia injury plane.

Custom nylon hobbles were created in-house. At week 0, a conventional dog harness was fitted and a nylon cuff was placed on one hind limb (side randomized) which was connected to the chest harness by interchangeable links that allow for a custom fit (Fig 1A). With proper adjustment, the hobble device restricted hind limb positioning so that the standing distance between fore and hind limb was approximately two-thirds the distance of an unrestrained animal. Additional adjustments to the hobbles were made accordingly as the animals increased in size to maintain the restricted hind-limb positioning. Hobbles were and kept in place for 8 weeks. Pigs underwent daily inspection for any sign of chafing and hobbles were adjusted accordingly. When in place, the hobble prevents full hip extension and pelvic lateral flexion in the transverse plane during gait.

### Fascia Injury

**Fig 2:**
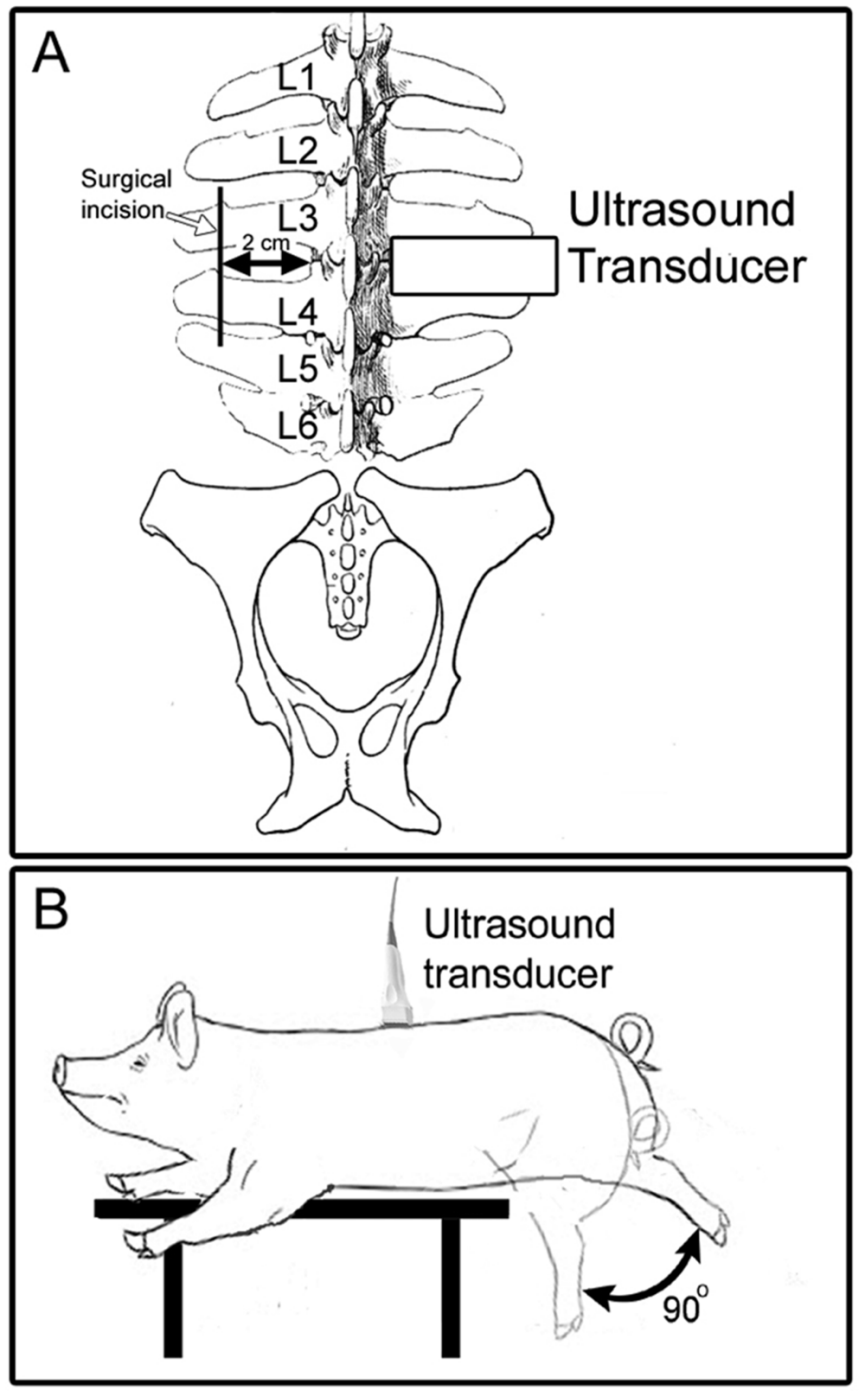
Ultrasound data acquisition methods. (A) Location of ultrasound images used for determination of fascia thickness. (B) Method used for acquisition of ultrasound cine-recording during passive trunk flexion.

At week 0, pigs in the injury groups underwent a unilateral fascia injury in the dorsal trunk (side randomized) at the L3-4 vertebral level 2 cm from the midline (Fig 1B). Anesthesia was induced by intramuscular injection of ketamine (20 mg/kg) and atropine (0.05 mg/kg) followed by maintenance with 4% isoflurane inhalation with 100% oxygen. Pigs were shaved and surgically prepared with betadine scrub and isopropyl alcohol. A 4 cm longitudinal skin incision was made 2.0 cm lateral to midline. Target depth of incision was the deep subcutaneous tissue layer between the subcutaneous membranous layer and the thoracolumbar fascia and great care was taken during the skin incision and closure to not penetrate the superficial layers of the thoracolumbar fascia. (Fig 1C). Blunt and microsurgical dissection tools were used to detach the perimuscular fascia from the adjacent deep subcutaneous tissue, producing a 4 cm x 4 cm injury centered 2 cm lateral to midline. The incision was closed with five interrupted nylon skin sutures and the animals were monitored daily. There were no wound infections among any of the groups. After 8 weeks of growth, the location of the surgical incision was ~2 cm from the lateral edge of the vertebral body, which was used as a landmark during ultrasound imaging (Fig 2A).

### Fascia Injury and Movement Restriction

At week 0, pigs randomized to injury plus hobble underwent the perimuscular microsurgical technique described above. On the day following the surgical procedure, a hobble device was fitted on the same side as the injury.

### Non-Intervention (Control)

Animals in the control group were fitted with a harness but not an ankle cuff and did not receive any surgical intervention or movement restriction.

### B-Scan ultrasound data acquisition

All ultrasound imaging was performed immediately after euthanasia by intravenous lethal injection of Fatal Plus (100 mg/kg) to prevent respiration artifact during imaging. Pigs were placed in the prone position on a surgical table. Ultrasound imaging was performed with a Terason 3000 (Terason, Burlington, MA. USA) scanner with a 4.0 mm, 10 MHz linear array transducer. Ultrasound images were acquired bilaterally at L2-3, L3-4 and L4-5 levels with the ultrasound probe oriented transversely, and the edge of the probe aligned with the lateral border of the vertebral body (Fig 2A). To measure tissue displacement within the connective tissue layers of the thoracolumbar fascia, ultrasound cine-recordings were acquired during passive flexion of the trunk (Fig 2B). After the ultrasound image acquisition described above, the pigs were repositioned so that the L4 level was at the edge of the table. The transducer was placed longitudinally on side of the dorsal trunk contralateral to the intervention (injury and/or hobble, with side randomized in control animals) at the level of the L3/L4 interspace, 2 cm from midline. A cine-loop (25 Hz frame rate) was captured over a 10 second period while the hips of the pig were manually flexed 90 degrees and returned to neutral position for 5 cycles at 0.5 Hz.

### Ultrasound image measurement of subcutaneous and perimuscular fascia tissue thickness

**Fig 3:**
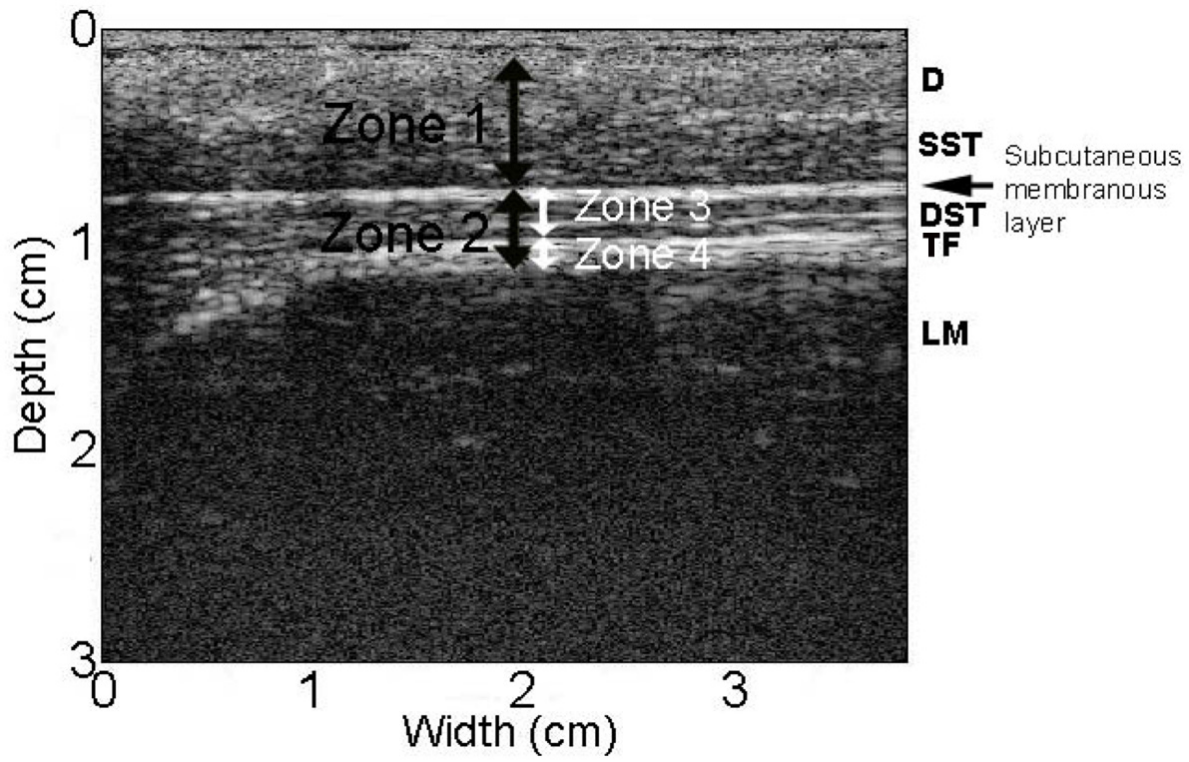
Location of tissue zones used for measurement of tissue thickness in ultrasound images. D: dermis, SST: superficial subcutaneous tissue, DST: deep subcutaneous tissue, TF: thoracolumbar fascia.

Ultrasound images on the injury side were reviewed to ensure consistency of intervention. All ultrasound measurements were performed on the side contralateral to the intervention (hobble and/or injury). This allowed examination of connective tissue remodeling away from the injury itself, with and without movement restriction. In control animals, the measured side was randomized. Ultrasound images were imported in Matlab and measured using a custom software program. In all images, the thickness of tissue layers was measured 2 cm from the lateral border of the vertebral body by identifying the following locations as shown in Fig 3: 1) superficial aspect of the dermis, 2) superficial edge of the subcutaneous membranous layer, 3) superficial edge of the thoracolumbar fascia, 4) superficial edge of the erector spinae muscle. The thickness of four discrete zones was calculated based on these measurements as illustrated in Fig 3.

### Measurement of thoracolumbar fascia shear strain

Ultrasound radio frequency (RF) data was captured and analyzed on the non-intervention side during the third flexion/extension cycle as previously described [7]. Subsequent analysis was conducted using an in-house program written in MATLAB (2010a, The MathWorks, Natick, MA USA). A 10x15 mm region of interest (ROI) encompassing the dermis, subcutaneous fat, fasciae layers, and muscle was identified. Shear strain was calculated in ten half-millimeter increments using a moving window starting 1 mm deep to the muscle boundary. The average shear strain was calculated among all the window positions and the average value was used for subsequent statistical analysis.

### Measurement of spinal cord substance P and CGRP

Spinal cord with attached dorsal root ganglia from levels L1-L5 were dissected immediately after death of the pig. The location of interest (L3) was marked with placement of suture, and immersion fixed in 4% PFA for 48 hours at 4°C. Samples were dissected while immersed in 0.1 M SPBS (sodium phosphate buffered saline, pH 7.4) into sections 1, 2, 3, and 4, with 1 being the most caudal. Samples were placed in 30% sucrose in 0.1 M SPBS for cryoprotection and stored at -20°C, then -80°C after taking samples for immunostaining. Spinal cord segment L3 was sectioned at a thickness of 40 μm on a freezing microtome. Substance P and CGRP immunostaining was performed using a 50-well plate. Antigen retrieval was done using 1% normal goat serum, 0.3% Triton X diluted in SPBS. Primary antibodies for substance P and CGRP (Phoenix Pharmaceuticals, Inc., Burlingame, CA) were diluted at 1:1000. Samples remained in primary antibody overnight on a shaker. Secondary antibody (Cy3 goat anti-rabbit, Jackson Immunoresearch, Inc., West Grove, PA) was diluted at 1:500. Samples were dried onto slides and rehydrated with SPBS before being covered. Samples were imaged with fluorescence miscroscopy (Olympus BX50 research microscope). Images were analyzed using Metamorph image analysis. A common threshold was used for each batch of images. Six square-shaped regions of dimension 50 microns x 50 microns were placed evenly along the areas of interest and values were logged, then averaged for the dorsal horn and lateral horn.

### Salivary cortisol measurements

Morning (8 am) and afternoon (4 pm) saliva samples were collected on a weekly basis using salivettes (Sarstedt, Numbrecht, Germany). Briefly, a cotton roll was placed in forceps and the pigs chewed on it, moistening it with saliva. The rolls were placed in the collection tubes and centrifuged for 2 minutes at 1000 x g. The saliva samples were frozen at -20C and batch analyzed using ENZO Cortisol EIA kits (cat. ADI-900-071) according to assay instructions.

### Statistical Analysis

Two-way analyses of variance were performed to examine the effects of movement restriction and injury on gait, ultrasound outcomes, substance P, CGRP, and cortisol measures. The model included terms representing the main effects of the two factors and their interaction. Repeated measures analysis of variance was used to compare weight among experimental conditions and across time. The model included three factors represented movement restriction, injury and time (0, 5 and 8-weeks), with the latter a within-subject factor. All analyses were done using SAS statistical software version 9.3 (SAS Institute, Cary, NC). Statistical significance was evaluated using α = .05.

## Results

**Fig 4:**
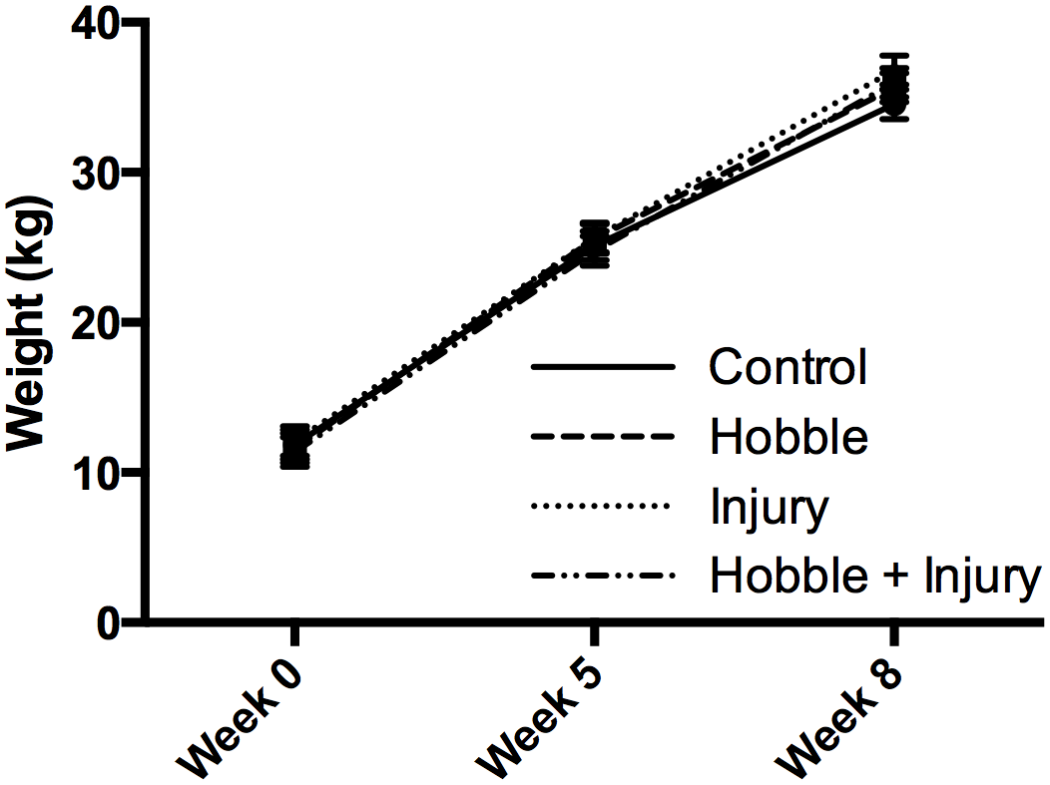
Pig growth over the course of the experiment. There were no significant weight differences among groups at 0, 5, and 8 weeks.

Pig weights were consistent among the four cohorts over the course of the study (Fig 4). There was a significant increase in weight over time (Repeated measured ANOVA, *F*(2, 32) = 983.65, *p* = < 0.001) across the 8 week period in all groups, consistent with a normal growth pattern. There were no significant weight differences among experimental groups at any of the time points (0, 5, and 8 weeks).

### Gait analysis

**Fig 5:**
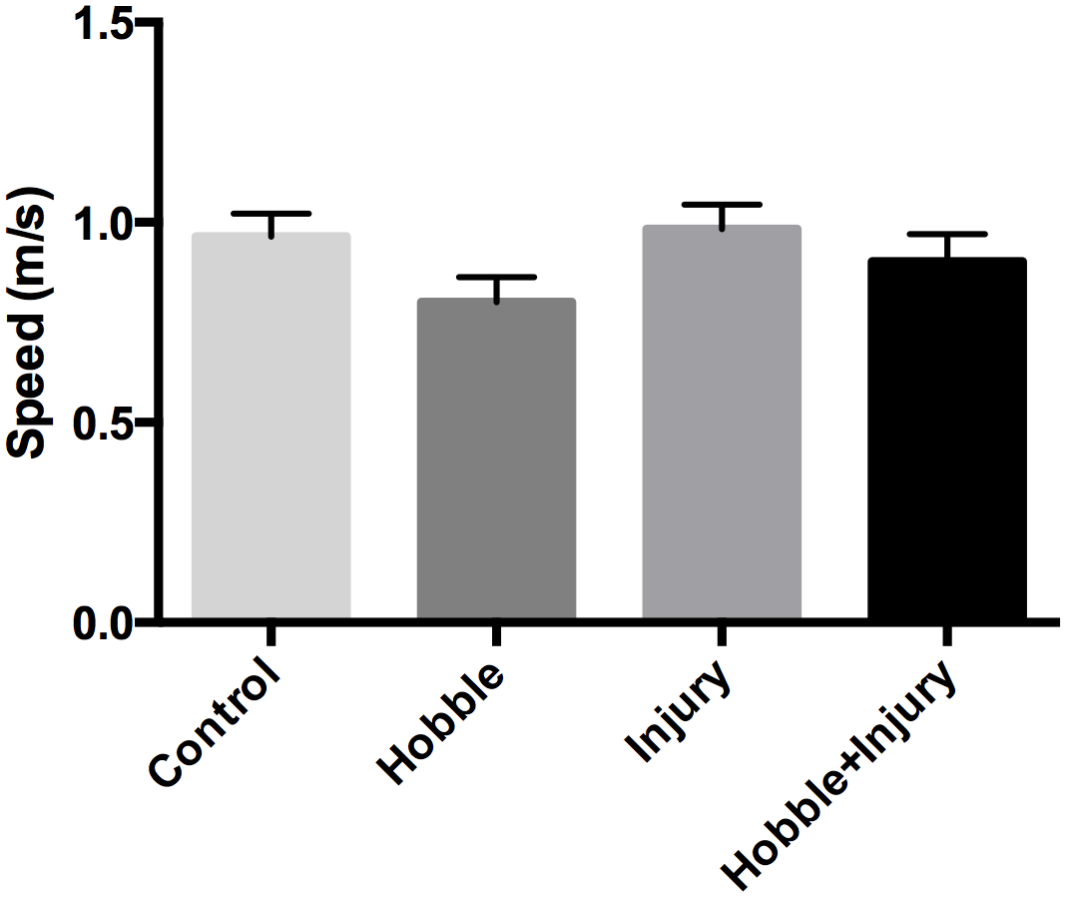
Gait analysis. Gait speed (m/sec) was measured at week 8. There was a significant main effect of movement restriction on gait speed *(ANOVA p* = .03), but no significant effect of injury (ANOVA *p = 2*.*56)*.

Motor function was assessed by gait speed, number of steps and stride length with hobble (if present) taken off. There was a significant main effect of movement restriction on gait speed (ANOVA, *F*(1, 15) = 5.51, *p* = .03), but no significant effect of injury (F(1,15) = 1.41, *p = 2*.*56*) (Fig 5).

### Ultrasound measurement of subcutaneous and perimuscular fascia tissue thickness

**Fig 6:**
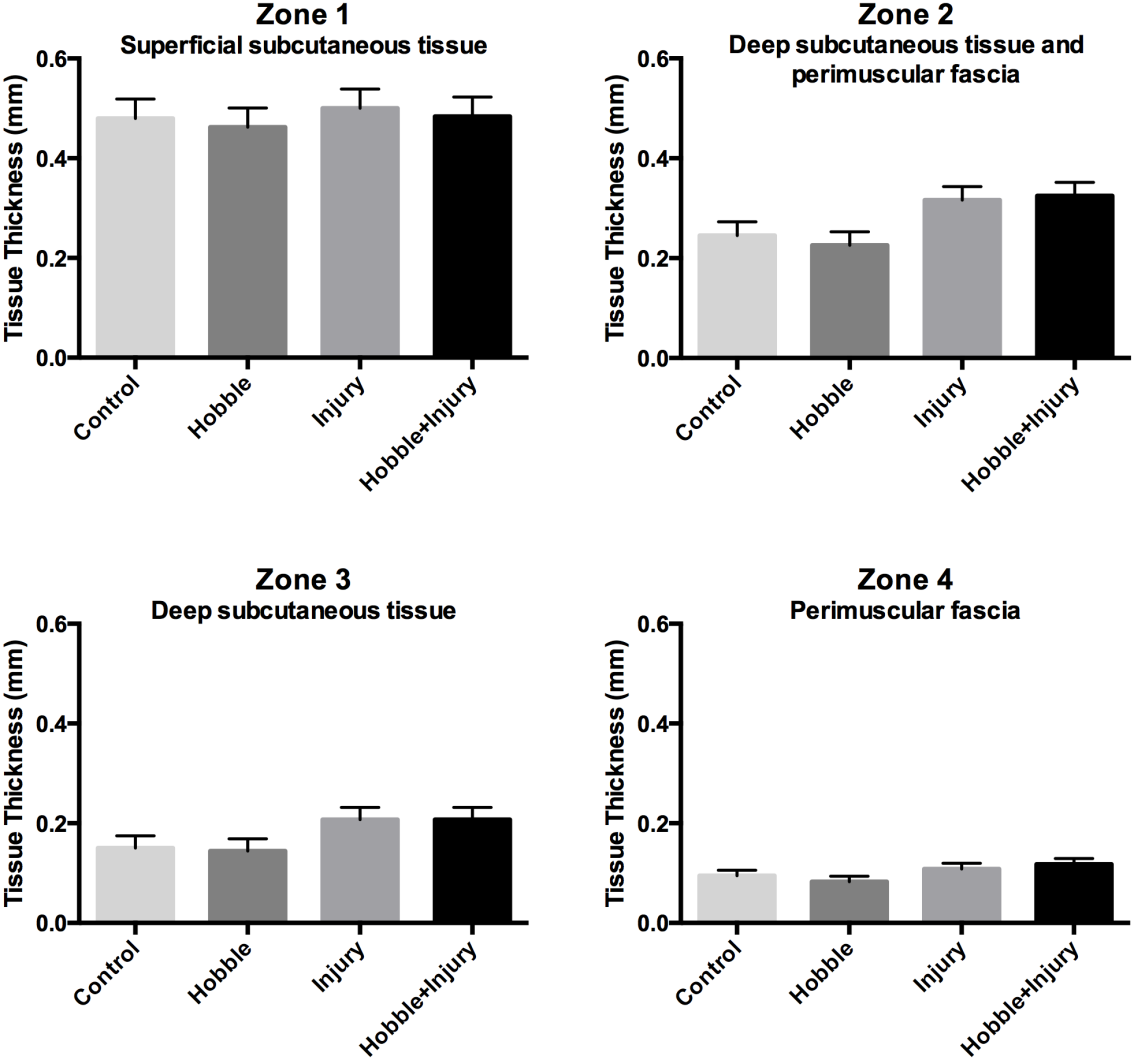
Ultrasound measurements of tissue thickness. The tissue thickness in four Zones (See Fig 3 for Zone locations) was measured at the L3-4 vertebral level on the non-intervention side. There was no significant difference in the combined thickness of dermis and superficial connective tissue (Zone 1) among groups (*p = 0*.*60)*. The thickness of Zone 2 (deep subcutaneous tissue and perimuscular fascia), Zone 3 (deep subcutaneous tissue) and Zone 4 (perimuscular fascia) all were significantly greater in the injured pigs compared with the other groups (ANOVA, main effect of injury for p = .007 (Zone 2), *p* = .026 (Zone 3) *p* = .04 (Zone 4)).

There was no significant difference in the thickness of dermis and superficial connective tissue (Zone 1) between groups (ANOVA, F(1,16) = 0.29, *p = 0*.*60*) (Fig 6). In contrast, the thickness of deep subcutaneous tissue and perimuscular fascia (Zone 2) at the L3-4 vertebral level was significantly greater in the injured pigs compared with the non-injured groups (ANOVA, main effect of injury *F*(1, 16) = 9.57, *p* = .*007*) (Fig 6). Additional analyses showed that both the deep subcutaneous tissue (Zone 3: *F*(1, 16) = 6.04, *p* = .026) and the perimuscular fascia (Zone 4: *F*(1, 16) = 4.80, *p* = .04) individually contributed to the increased Zone 2 tissue thickness in the injured animals (Fig 6). A similar pattern was observed at the L-2-3 and L4-5 vertebral levels, but differences between groups were not statistically significant.

### Ultrasound measurement of thoracolumbar fascia mobility (shear strain)

**Fig 7:**
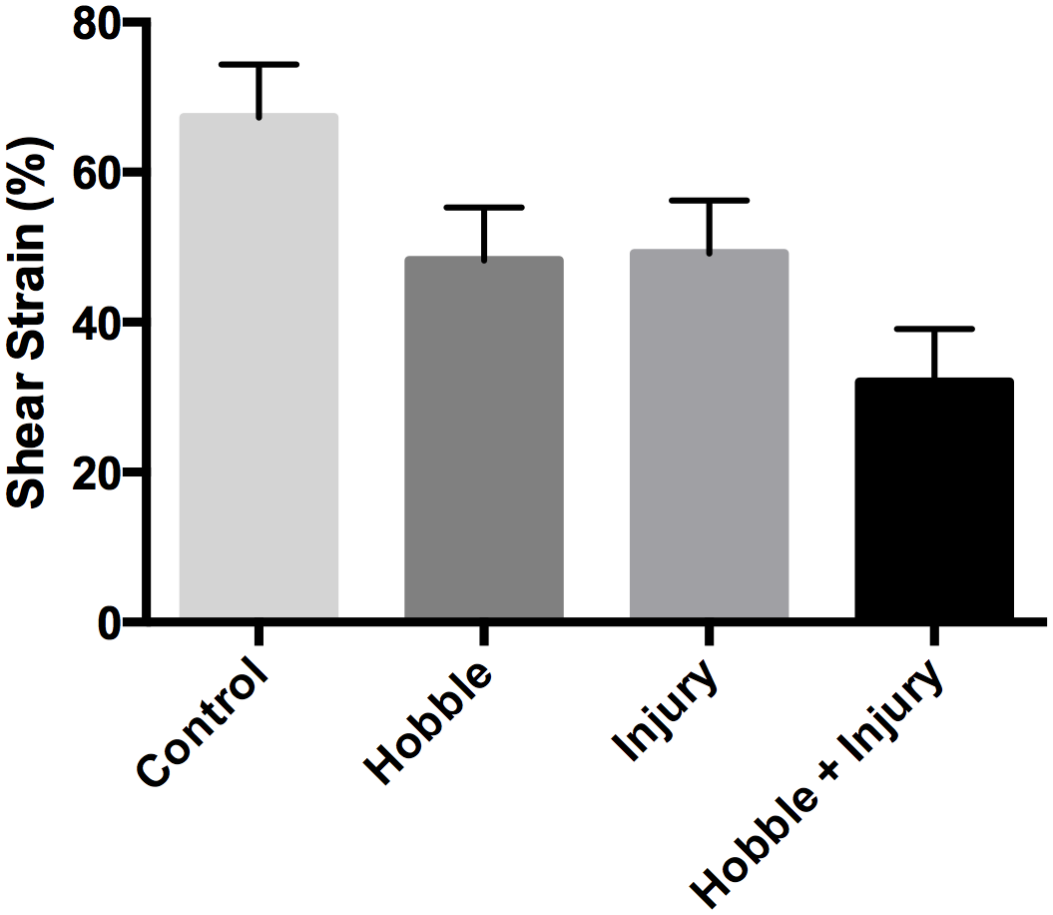
Perimuscular fascia shear strain measurements. Both injury and movement restriction (hobble) led to a significant reduction in thoracolumbar fascia shear strain (ANOVA main effects of injury *p* = .027, and hobble *p* = .021). There was no significant interaction between the effects of injury and hobble.

Both injury and movement restriction (hobble) led to a significant reduction in thoracolumbar fascia shear strain (ANOVA main effects of injury *F*(1, 16) = 5.86, *p* = .027, and hobble *F*(1, 16) = 6.52, *p* = .021) (Fig 7). There was no significant interaction between injury and hobble with the combined injury plus hobble group showing additive effects of the two factors on fascia mobility. There additive effects resulted in a 52% reduction in shear strain compared to controls (compared with a 28% reduction for movement restriction alone).

### Spinal cord dorsal horn Substance P and CGRP expression

In both the medial and lateral dorsal horn, there were no significant differences in either Substance P or CGRP immunoreactivity between groups (ANOVA, Substance P in dorsal horn F(3,13) = 0.49, *p* = .*70*, lateral horn F(3,13) = 0.36, *p* = .*78*, CGRP in dorsal horn F(3,14) = 0.23, *p* = .*87*, lateral horn F(3,14) = 0.66, *p* = .*59*).

### Salivary cortisol measurements

No significant differences in AM or PM cortisol levels were found between groups (ANOVA for AM cortisol F(3,17) = 0.48, *p* = .*70*, PM cortisol F(3,17) = 0.39, *p* = .*76*).

## Discussion

In pigs, a combination of fascia injury and movement restriction produced increased fascia thickness and decreased mobility in connective tissue layers similar to those observed in a study of humans with chronic LBP [6, 7]. We found no significant differences in spinal cord dorsal horn Substance P or CGRP among groups, suggesting that none of the experimental conditions induced severe chronic pain. It is possible that the injury or hobble could have caused discomfort or pain not severe enough to cause spinal cord neuroplastic changes detectable using our methods, or that the expression of these neuropeptide could have been altered at earlier or later time points. Importantly, however, because the reductions in fascia mobility found in this study were not accompanied by spinal cord neuroplastic changes suggesting chronic pain, our results do not support the notion that loss of shear plane mobility directly alters nociceptive input from the fascia. This is also consistent with our previous results in humans showing a lack of significant correlation between thoracolumbar shear strain and pain symptoms [7]. On the other hand, restricted fascia mobility may cause altered proprioception and movement patterns, and thus could be involved in low back pain pathophysiology without being the direct source of nociceptive input.

Injury alone caused both a significant increase in fascia thickness and decrease in fascia mobility on the non-injured side, demonstrating the presence of a pathological process involving the thoracolumbar fascia extending beyond the injured area. Movement restriction alone, on the other hand, did not change fascia thickness but did decrease fascia shear plane mobility, consistent with the formation of connective tissue adhesions due to chronically reduced movement [25]. The combination of injury plus movement restriction had additive effects on fascia mobility. This suggests that adding movement restriction to a soft tissue injury can worsen an already increased tendency of fasciae to adhere together and lose shear plane mobility.

It is well known clinically that restricting movement of joints causes adhesions in periarticular connective tissue, especially after an injury or surgery [26, 27]. Our previous studies in humans, and the results of our porcine model, indicate that a similar pathology can occur in the fasciae of the dorsal trunk in response to a mild fascia injury, especially in the presence of movement restriction. Although back “sprains” are a common occurrence, we currently have no diagnostic method to measure their extent or impact. Our results suggest that a back injury involving fascia, even when healed, can affect the relative mobility of fascia layers in tissues on the other side of the back that were not immediately involved in the initial injury, especially when movement is restricted. Our measurements at the L2-3 and L4-5 levels showed similar trends to those observed at the L3-4 level (increased thickness and decreased mobility), however these did not reach statistical significance at the L2-3 and L4-5 levels. The reason for this may be technical, as the connective tissue planes are at their flattest and most parallel to the skin in the middle of the back (L-3-4 level) and these are the optimal conditions for making our measurements. The presence of slight curves in the connective tissue planes at L2-3 and L4-5 may have introduced some additional variability in the data that obscured any differences that may have been present between groups. We are therefore not able to make a statement at the present time as to whether our findings represent local or generalized connective tissue changes in the back.

The injury in our porcine model was intended to produce a mild tear between the membranous subcutaneous layer and the perimuscular fascia through a small skin incision. It is interesting that the deep tissue layers involved on the side contralateral to the injury are the equivalent layers involved in humans with LBP (deep subcutaneous layer and perimuscular fascia). In contrast, even though the superficial subcutaneous layer was involved in the injury in the porcine model, we found no evidence that this layer was affected on the non-injured side, and the equivalent layer was similarly unaffected in the human subjects with LBP.

Injury alone did not affect gait speed, compared with controls, which is consistent with an injury that has healed over the 8 week period without causing functional impairment. In contrast, hobbled animals had decreased gait velocity, even with the hobble taken off. This suggests that the effect of the hobble on gait mechanics was more pronounced than the effect of the injury. Nevertheless, movement restriction alone did not increase fascia thickness, as an injury was needed to produce the combined pathology (increased thickness and decreased mobility) seen in humans with LBP.

Our initial study using this porcine model did not intend to investigate specific pathological mechanisms, but rather aimed at using ultrasound as a translational tool to determine whether clinically relevant pathology could be produced experimentally. A notable difference between our human and porcine studies is that our perimuscular fascia shear plane measurements were made 10 minutes post-mortem in pigs, which had the advantage of eliminating brain and spinal cord mediated reflex muscle activity, including breathing, while still allowing us to measure the structural and biomechanical behavior of fascia.

An inevitable limitation of any animal model in relation to human low back pain is the animal’s quadruped posture, compared with the biped posture of humans. Consideration of biomechanical differences between humans and animals is especially important in studies of the spine and intervertebral discs due to the different gravitational loads on the spine in biped vs. quadruped posture [28]. However, the structural organization and function of the trunk musculature and associated fasciae are essentially conserved with little alteration between quadruped mammals and humans [29] [30]. This includes the thoracolumbar fascia whose principal biomechanical function is to transfer loads from the upper spine and arms to the pelvis and legs during walking which applies both to biped and quadruped gait.

A further limitation of our study is that the study period (2 months) was shorter than the duration of LBP in our previous human study (greater than 12 months). Due to practical considerations, we were not able to study fully-grown animals, since skeletal maturity in domestic pigs is 12–14 months at which point the pigs would have been ~200 kg by the end of the experiment which would not have been manageable, and thus the pigs were growing while the experiment was taking place, which is different from the adult population that was studied in our LBP study. Nevertheless, we did see pathology relevant to that observed in humans and thus extending this model further in time may be useful in future studies.

In conclusion, a porcine model combining thoracolumbar fascia injury and movement restriction for 8 weeks resulted in increased thickness and decreased mobility of the thoracolumbar fascia similar to that observed in human subjects with chronic LBP. However, the application of these findings to people with chronic low back pain must be carefully considered, especially given our lack of evidence of differences in spinal cord nociceptive neuropeptides between experimental groups. Our results do, on the other hand, suggest that a back injury involving fascia, even when healed, can affect the relative mobility of fascia layers in tissues on the other side of the back that were not immediately involved in the initial injury, especially when movement is also restricted. Future studies will be needed to examine the mechanisms responsible for these abnormalities, and their potential reversibility in response to treatment.
